# Visual laterality in dolphins: importance of the familiarity of stimuli

**DOI:** 10.1186/1471-2202-13-9

**Published:** 2012-01-12

**Authors:** Catherine Blois-Heulin, Mélodie Crével, Martin Böye, Alban Lemasson

**Affiliations:** 1 UMR 6552 University of Rennes 1 - CNRS, Station Biologique, 35380 Paimpont, France; 2 Département scientifique, Planète Sauvage, 44710 Port-Saint-Père, France

## Abstract

**Background:**

Many studies of cerebral asymmetries in different species lead, on the one hand, to a better understanding of the functions of each cerebral hemisphere and, on the other hand, to develop an evolutionary history of hemispheric laterality. Our animal model is particularly interesting because of its original evolutionary path, i.e. return to aquatic life after a terrestrial phase. The rare reports concerning visual laterality of marine mammals investigated mainly discrimination processes. As dolphins are migrant species they are confronted to a changing environment. Being able to categorize new versus familiar objects would allow dolphins a rapid adaptation to novel environments. Visual laterality could be a prerequisite to this adaptability. To date, no study, to our knowledge, has analyzed the environmental factors that could influence their visual laterality.

**Results:**

We investigated visual laterality expressed spontaneously at the water surface by a group of five common bottlenose dolphins (*Tursiops truncatus*) in response to various stimuli. The stimuli presented ranged from very familiar objects (known and manipulated previously) to familiar objects (known but never manipulated) to unfamiliar objects (unknown, never seen previously). At the group level, dolphins used their left eye to observe very familiar objects and their right eye to observe unfamiliar objects. However, eyes are used indifferently to observe familiar objects with intermediate valence.

**Conclusion:**

Our results suggest different visual cerebral processes based either on the global shape of well-known objects or on local details of unknown objects. Moreover, the manipulation of an object appears necessary for these dolphins to construct a global representation of an object enabling its immediate categorization for subsequent use. Our experimental results pointed out some cognitive capacities of dolphins which might be crucial for their wild life given their fission-fusion social system and migratory behaviour.

## Background

Laterality, previously considered to be exclusively a human characteristic, has been show in many vertebrates as well as invertebrate species (for example: [1-6]). Thus, the asymmetry of cerebral functions seems to be the rule rather than the exception in the animal kingdom [7]. Analyses of lateralized motor, perceptual or behavioral responses broaden our understanding of cerebral organization and of treatment of information by each cerebral hemisphere.

Characteristics of perceived stimuli can be linked to the treatment of the information received by one of the cerebral hemispheres and to the implication of a given hemisphere. This link is modulated by subjects' internal state such as levels of hunger, vigilance or stress [8], as well as their age [9] or social environment [10]. Moreover, stimulus characteristics like emotional value [11,12] and novelty [13,14] are known to influence perceptual laterality.

The anatomical characteristics of the visual nervous system make visual laterality a good candidate to study perceptual laterality, and most particularly in dolphins. It is well known that the two cerebral hemispheres do not receive sensory information from a single stimulus in the same proportions. For instance in many mammals, the contralateral hemisphere receives monocular visual information faster (crossed fibers) than that received by the ipsilateral hemisphere (uncrossed fibers) [8,15]. So the involvement of each cerebral hemisphere depends on the organization, the quantity and the transmission speed of nervous impulses [15]. Dolphins are particularly interesting model for hemispheric laterality investigations for at least four reasons. First, their eyes are situated laterally and all their optic fibers cross [16,17] allowing a reliable interpretation of the underlying cerebral asymmetry. The left hemisphere receives visual information exclusively from the right eye, and the right hemisphere from the left eye. Moreover, stronger isolation of brain hemispheres is due to relatively less developed *corpus callosum *[18]. Preferential use of a given eye (also called eye use bias) at the group level indicates the treatment of visual information by the contralateral hemisphere [11,19]. Second, several reports evidence the existence of lateralized behavior in marine mammals (whales: [20,21]; dolphins: [16,22-25]). For example, the general tendency of dolphins to swim counter-clockwise [16,24,25] might indicate the presence of laterality in this species. Third, previous reports indicated that dolphins generally use monocular vision (due to a narrow binocular visual field), and that they visually solve visio-spatial tasks better using their right eye and therefore their left brain hemisphere [17,24,26-29]. However, a recent study has shown that even if dolphins were clearly able to visually discriminate between familiar and unfamiliar human beings, they preferentially used their left eye to look at those two categories of people [30]. Forth, dolphins possess a high brain/body ratio [31], and several of the brain's morphological traits differ from those of terrestrial mammals [31-34]: their brains present anatomical structures similar to those of highly evolved brains, but at the same time, the organization of their rudimentary neocortex recalls more that of the hypothetical ancestor of mammals [35]. Analysis of their perceptual laterality should help understand the implication of each hemisphere in the treatment of various types of information, compared to other species.

One of the most frequently studied characteristics of stimuli influencing perceptual laterality is novelty). A certain consensus appears concerning the processing of the novelty of a stimulus ('Novelty hypotheses'). Generally, the left eye is privileged to look at novel stimuli (chicks: [36-38], fish: [39,40]) and/or to be better at processing or storing visual information which allows recognition of individual conspecific [41,42]. But exceptions can be found. De Boyer Des Roches et al [43] have shown that mares used preferentially their right eye to explore novel objects. According to Navon [44,45], characteristics of objects can be visually analyzed either globally or in detail (local traits). Local characteristics are mainly analyzed by the posterior superior temporal-parietal regions of the left hemisphere while global characteristics are analyzed by the posterior superior temporal-parietal regions of the right hemisphere ('Information treatment modality hypotheses') [46-51]. Thus, a link between the type of information treatment and the stimulus characteristics can be surmised: global analysis should be favored when observing a familiar object, whereas local analysis should occur in presence of a novel object to gather detailed information.

Our aim was to understand how these marine mammals perceive, analyze and treat visual stimuli with different levels of familiarity (i.e. unfamiliar, familiar non-manipulated, very familiar manipulated). In this study the following questions were investigated experimentally: (1) Is there a behavioral laterality in dolphins, as it was found in others marine animals? (2) How does stimulus novelty affect the visual preference in dolphins? Three contradictory predictions can be done. First, based on the 'novelty hypotheses', dolphins should preferentially use their left eye to look at novel objects (unfamiliar) and should not display any preference for non-novel objects (familiar and very familiar). Second, according to the 'Information treatment modality hypothesis' [44,45], dolphins should use their left eye to look at very familiar objects, because a global visual inspection is sufficient to recognize and categorize the stimulus, but use their right eye to look at unfamiliar objects for which a more detailed visual inspection is required. The third prediction was based on Thieltges et al [30] results. These authors have demonstrated that dolphins used their left eye to look at human whatever their degree of novelty. So we wondered whether dolphins would generalize this left eye preference to look at all kinds of objects, regardless of their degree of novelty. 3) Is there a link between visual and swimming laterality? As swimming laterality seems strong in captive dolphins, we also analyzed the swimming preferences of our animals in order to control that the visual laterality observed was not a direct consequence of swimming preferences.

## Results

### Reactivity

Stimulus category neither influenced the number of trials when dolphins approached and observed the set-up spontaneously (Friedman test, df = 2, χ^2 ^= 6.35, p = 0.096), nor the total number of gazes at objects of each category significantly (Friedman test, df = 2, χ^2 ^= 5.08, p = 0.166). Dolphins preferred monocular to binocular vision to look at object of all categories (Wilcoxon tests, z = 2.02, p = 0.043).

### Laterality

#### First reaction (Table 1)

Three dolphins (Cecil, Peos and Thea) used their left eye to observe very familiar previously manipulated objects, whereas two dolphins (Cecil and Mininos) used their left eye to observe familiar never-manipulated objects. None of the dolphins was lateralized when viewing unfamiliar objects (Table 1).

**Table 1:** Variation of the laterality index (IVL) according the level of object familiarity for the first reaction, p: binomial test, bold character: significant results, p < 0.05, L: left eye used

	CECIL	PEOS	MININOS	THEA	AMTAN
**Familiar-Manipulated**					
VLI	**-0.88**	**-0.58**	-0.29	**-0.63**	-0.29
P	**0.001**	**0.019**	0.424	**0.021**	0.332
Bais	**L**	**L**	ns	**L**	ns

**Familiars-Never Manipulated**					
VLI	**-0.56**	-0.47	**-0.83**	0.07	0.06
P	**0.031**	0.118	**0.006**	1.000	1.000
Bais	**L**	ns	**L**	ns	ns

**Unfamiliar**					
VLI	0.29	0.18	0.37	0.43	0.33
P	0.424	0.629	0.167	0.180	0.238
Bais	ns	ns	ns	ns	ns

**Figure 1:**
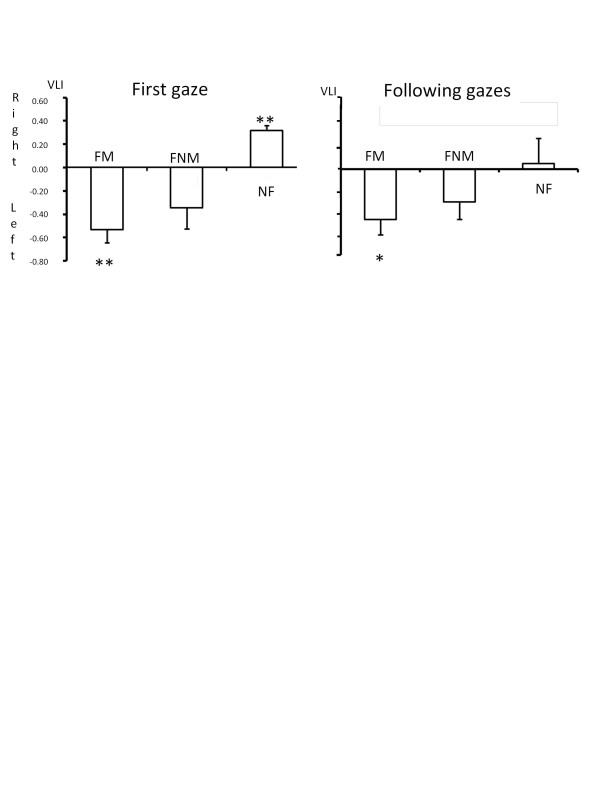
**Visual laterality index (VLI) in relation to stimulus category**. FM: familiar, previously manipulated objects, FNM: familiar, never manipulated objects, UF: unfamiliar objects. Stars: results of t test, _*_: p < 0.01, _**_: p < 0.002.

At the group level, a significant left bias was evident when watching at very familiar previously manipulated objects (t test, t = -4.80, *df *= 4, p = 0.009) (Figure 1A). Conversely, a significant right bias was evidenced for watching at unfamiliar objects (*t *test, t = 7.50, *df *= 4, p = 0.002). When watching at familiar never manipulated, objects the dolphins did not use one eye preferably (*t *test, t = -1.95, *df *= 4, p = 0.123) (Figure 1A).

Strength of laterality did not vary significantly with experimental situation (Friedman test, ABS(VLI), χ^2^= 1.20, *df *= 2, p = 0.55).

#### Reactions following the first reaction

Peos, Mininos and Thea used more their left eye to look at very familiar, previously manipulated, objects. Two dolphins (Cecil and Peos) used their left eye significantly more frequently to look at familiar never manipulated objects. Conversely, Amtan used more her right eye to observe unfamiliar objects (Table 2).

**Table 2:** Variation of the laterality index (IVL) according the level of object familiarity for the reaction following the first reaction, p: binomial test, bold character: significant results, p < 0.05, L: left eye used

	CECIL	PEOS	MININOS	THEA	AMTAN
**Familiar-Manipulated**					
VLI	-0.28	**-0.61**	**-0.67**	**-0.78**	-0.03
P	0.132	**0.000**	**0.000**	**0.000**	1.000
Bais	ns	**L**	**L**	**L**	ns

**Familiars-Never Manipulés**					
VLI	**-0.48**	**-0.60**	-0.50	-0.25	0.29
P	**0.003**	**0.035**	0.146	0.454	0.332
Bais	**L**	**L**	ns	ns	ns

**Unfamiliar**					
VLI	**-0.69**	0.10	-0.14	0.25	**0.73**
P	**0.000**	0.711	0.511	0.454	**0.007**
Bais	**L**	ns	ns	ns	**R**

At the group level, as for the first reaction, a significant left bias was evidenced for watching at very familiar previously manipulated objects (*t *test, t = -3.41, *df *= 4, p = 0.027), but no significant bias was found for watching at familiar never manipulated objects (*t *test, *df *= 4, t = -1.91, p = 0.129). The following reactions to unfamiliar objects differed significantly from the first reaction to these objects as no bias towards the usage of the left or right eye was found (*t *test, *df *= 4, t = 0.23, p = 0.832) (Figure 1B).

Strength of laterality did not vary significantly between the three experimental situations (Friedman test, χ^2 ^= 0.89, *df *= 3, p = 0.85).

#### Comparisons of bias and strength of laterality between the first and the following reactions

Laterality indices and strength of laterality did not differ significantly between the first and the following reactions irrespective of the experimental situation (Wilcoxon test, n = 5, FM situation: VLI z = 0.13 p = 0.893, Abs (VLI) z = 0.13 p = 0.893; FNM situations: VLI z = 0.40 p = 0.686, Abs (VLI) z = 0.40 p = 0.686; NF condition: VLI z = 1.21 p = 0.225, Abs (VLI) z = 0.40 p = 0.686).

#### Rotational bias

**Figure 2:**
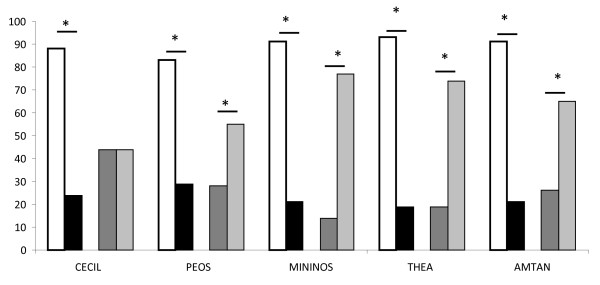
**Swimming categories: numbers of observations of each swimming type for each dolphin**. White bars: all rotations; black bars: swimming without direction; dark grey bars: clockwise rotation, light grey bars: counter-clockwise rotation. *: results of binomial test p < 0.05.

All our dolphins were strongly lateralized, they swam significantly more frequently in one direction than without direction (binomial test: lateralized versus no direction, p < 0.001). Four of the five dolphins preferred to swim counter-clockwise (Figure 2). At the group level our dolphins also showed a preference to swim counter-clockwise (*t *test RI, t = -3.39, *df *= 4, p = 0.027).

## 4. Discussion

Our data showing that all our dolphins used monocular vision spontaneously more frequently than binocular vision confirmed previous reports [26]. This is certainly due to the lateral eye position determining a small binocular visual field in this species. A bias to use their left eye (right hemisphere) to look at very familiar previously manipulated objects emerged clearly at the group level for both the first gaze and the following reactions. The first gaze reaction to unfamiliar objects revealed a preference to use the right eye (left hemisphere). However, this bias disappeared during the subsequent reactions. No eye preference was found for familiar never manipulated objects. Our dolphins also showed a counter-clockwise bias when swimming.

We can first confirm that our dolphins were strongly lateralized when swimming and when looking at objects. The fact that very familiar objects were visually inspected with the left eye while unfamiliar objects were visually inspected with the right eye, support the first of our three predictions based on the information treatment modality highlighted by Navon [44,45].

This difference of laterality at the group level between previously manipulated stimuli (use of left eye) and unfamiliar stimuli (use of right eye) might thus be explained by the way information leading to identification of objects is treated. During the first inspection of unfamiliar stimuli, dolphins might analyze details of the objects notably to build a spatial mental representation in three dimensions of the different parts of the object. This type of analysis is characteristic of information treatment by the left hemisphere [46,50,51]. Conversely, visual analysis of very familiar objects remains global and is characteristic of information treatment by the right hemisphere. Our results confirm the difference between treatments: dolphins used their left eye (right hemisphere) to look at very familiar objects (global analysis) and their right eye (left hemisphere) to look at unfamiliar objects (local analysis). No eye preference was found for the inspection of objects presenting an intermediate familiarity value (familiar non manipulated objects). However, as soon as a previously unfamiliar object has been seen once, this object becomes familiar, but still not manipulated, so no more eye preference is found in the subsequent visual inspections. This change from using the left hemisphere when treating visual information of an unknown shape to using the right hemisphere when that shape has become familiar has been reported previously [48]. The preference to use the right eye to explore details of objects confirm previous reports on the visual discrimination capacities of dolphins [17,26-29]. Dolphins discriminated visually better and solved visio-spatial tasks better using their right eye.

This rapid switch of classes from unfamiliar to familiar support studies that concluded in favor of a rapid visual memorization in dolphins [52,53]. The difference of treatment between very familiar and familiar objects also highlights the importance of the manipulation in dolphins. We can suppose that dolphins may possibly have difficulties in constructing a spatial mental representation of familiar never manipulated objects and of unfamiliar objects that have become familiar solely through visual modalities. If this is the case, manipulation of objects by dolphins could facilitate their construction of a global representation of an object enabling dolphins subsequently to categorize it directly, as echolocation and vision are generally linked [53-56].

In a previous study with the same dolphins we found a left-eye preference to look at familiar and unfamiliar human beings [30]. This shows that any human being is treated visually as are very familiar objects, a global inspection being sufficient to have a mental representation of this kind of stimulus. We must acknowledge that a human observer was present in this study near the exposed object and that this could have influence the visual response of our dolphins. However, the observer was the same person for all object presentations and always stood at the same place. The fact that the eye used changes with the object category let us think that the presence of the observer had no impact on our data.

In addition to perceptual laterality, we also analyzed swimming laterality and could thus control that the visual laterality observed here was not a direct consequence of swimming preferences. Our dolphins were lateralized at the motor level. Our group revealed a preferred rotation direction (counter-clockwise), thus supporting most previous reports on this theme [16,24,25]. This verified our third hypothesis. This rotational bias alone cannot explain the predominance of the use of one eye since the direction of their visual laterality is influenced by stimulus category.

As dolphins are migrant species they are confronted to a changing environment. Being able to categorize new versus familiar objects allows dolphins a best and a rapid adaptation to novel environment. Since we have demonstrated that dolphins used more their left eye to inspect well-known objects (this paper) as well as humans [30], future investigations should explore about the visual preference of dolphins when looking at conspecifics. As dolphins are social species and as other cetacean species, like beluga whale (calves were more on the right side of their mother) [21], expressed social laterality, it will be legitimate to wonder whether dolphins will also use their left eye to look at all kinds of congeners or if the familiarity with the conspecifc has some importance. This social laterality at population level could play an important role in the daily life of species like bottlenose dolphins presenting a fission-fusion social system, i.e. with opportunities to meet conspecifics of different degrees of familiarity.

## Conclusion

We clearly show the influence of a familiarity gradient on dolphins' visual laterality. A bias to use their left eye (right hemisphere) to look at familiar previously manipulated objects emerged clearly at the group level, whereas we found a bias to use the right eye (left hemisphere) to look at unfamiliar objects. A global vs detailed visual treatment can explain the observed laterality.

Swimming laterality was strong but no link was found between visual and swimming laterality. So our experimental results pointed out some cognitive capacities of dolphins that are indispensible for their wildlife [52]. To complete our studies, an analysis of social laterality will be pertinent. On which side a dolphin approach another congener and can the familiarity with the congener modulate this laterality?

## Methods

### Subjects

Our subjects were five captive-born dolphins: three 5 to 25-year old males (Mininos, Peos and Cecil), and two females, respectively 8 and 17 years old (Amtan and Thea). They were housed by the Cité Marine at the Planète Sauvage Safari Park (44, France) in a large aquatic facility (4 pools with 8.5 million liters of water). Six human caretakers fed the dolphins with fish, 6 to 8 times a day, during training and public presentations.

Veterinary assessment of these dolphins' vision revealed no deficiencies and no pathology that might bias our research. The dolphins participated in six training session every day, between 10 o'clock and 16'clock. They never participated in a public presentation during the course of this study.

## Methods

The dolphins were tested in the round, 20 m in diameter, 4.85 m deep maximum, nursery pool. Three types of stimulus were presented: familiar, previously manipulated objects (balls, discs...) (FM), familiar never manipulated objects (diving mask, watering can, boots...) (FNM) and unfamiliar objects (unknown: storage box, kettle, cycle helmet, thermos flask...) (UF). The size range of dolphin's toys varying in size (0.2 m to 1 m), as well as in shape and color. Each stimulus was presented once. Twenty objects of each category were used to test the dolphins and each stimulus was presented once.

Before a stimulus was presented, it was hidden behind a curtain so that the dolphins could not see it. The curtain was suspended from a 2 m high, 1.20 m wide PVC frame with which the dolphins were already familiar [56]. This curtain was placed 1.20 m from the pool edge. Behind the curtain the object was placed on a wooden box (35 × 40.5 × 45.5 cm) with which the dolphins were also familiar. This box was used to raise the stimulus making it easier to see (preliminary experiment). The stimuli were presented in air for practical reasons and can be done because dolphins' visual acuteness is globally similar in above and below the water surface [57-59]. Moreover, underwater dolphins might have used not only vision but also echolocation. This frame + box were put in place, two minutes before a test. A trial started when the experimenter opened the curtain. The object remained visible for three minutes. No previous training was necessary, and no reinforcement was given. Dolphins were observed during 180 minutes (20 objects of each category × 3 categories × 3 minutes per object).

During a presentation, all the reactions of the dolphins to the experimental set-up at the surface were recorded, but observations were restricted to the half of the pool close to the objects (20 m × 9 m) [30]. So our laterality study only concentrated on short-distance visual inspection, which means that all dolphins had the chance to first see the object from far before approaching, a still reasonable distance to appreciate whether the object was novel or not. The subjects were filmed in this area of the pool by a video-camera (JVC Everio GZ-MG332HE) placed on top of the frame [30]. The observer, standing randomly either to the left or to the right of the curtain, described, using a dictaphone (SCOTT DVR 500), all the reactions observed in the area not covered by the camera. The following variables were recorded:

• The identity of the dolphins in the area

• The total number of short gazes at the water surface and the eye with which the subject perceived the stimulus. We first distinguished short and long gazes (i.e. lasting respectively less and more than 2 seconds) (Kuczaj, Pers. Com.). But dolphins performed too few long gazes to possibly include them in the analysis.

At least 15 minutes elapsed after a feeding session, an interaction with careers or a previous test before a test. Three to eight (mean 5.71) tests were made per day and occurred at various time of the day (between 10 h and 18 h).

### MC conducted a blinded analysis of videos

Independent of experimental tests, the direction of dolphins' spontaneous rotations in the pool was investigated. Hourly instantaneous scan observations between 9.30 o'clock and 16.30 o'clock recorded either rotation direction (clockwise or counter-clockwise) or the absence of rotation for each dolphin. Eight scans were performed per day, yielding 112 scans.

### Statistical analyses

Friedman tests analyzed the numbers of trials when a subject approached the objects of a given category spontaneously in order to look at them and the numbers of gazes at each object. Wilcoxon tests compared use of monocular vision to use of binocular vision.

The first reaction of each dolphin, i.e., the first time a subject observed the object and all the reactions following, the first reaction (= following reactions in the text), were considered separately to estimate laterality. Thus, a visual laterality index (VLI) was calculated for each dolphin using the formula: (R-L)/(R+L), R and L are the numbers of times the right eye and the left eye were used to observe objects. This index varies from -1 to +1, negative values indicate a preference to use the left eye and positive values a preference to use the right eye. In addition, the absolute value of VLI estimates strength of laterality. Binomial tests evaluated each subject's preference to use one eye by comparing the numbers of times the right eye and the left eye were used for each experimental category. At the population level, *t *tests, applied to VLI values, estimated the presence of laterality at the group level. Friedman tests on VLI values and on their absolute values evaluated variations of direction and of strength of laterality between experimental conditions. Wilcoxon tests compared direction and strength of laterality between the first reaction and the following reactions.

Finally, binomial tests compared 1) the number of times a subject's swimming presented a lateralized rotation direction to the number of times its swimming presented no precise rotation direction, and 2) the number of times it swam clockwise to the number of times it swam counter-clockwise. A rotation index (RI): (number of times a subject swam clockwise - number of times it swam counter-clockwise)/(total number of rotations) was calculated for each subject. A *t *test on these RI values estimated the presence of a preferred direction at the group level.

Significance level for all statistical analyses was p < 0.05.

Experimental research reported in this manuscript has been performed with the approval of ethics committee (DDSV n° 04672).

## Authors' contributions

MC participated in the design of the study, conducted the experiments, and performed the video and statistical analysis. MB participated in the design of the study and conducted the experiment. AL initiated the study, participated in the design of the study and participated in the preparation of the manuscript. CBH initiated the study, contributed to the design of the study, participated in the statistical analysis, and in the preparation of the manuscript. All authors have read and approved this manuscript.
